# 4-(2-Fluoro­benzo­yl)-1-[2-(4-hy­droxy­phen­yl)-2-oxoeth­yl]piperazin-1-ium trifluoro­acetate

**DOI:** 10.1107/S1600536812042857

**Published:** 2012-10-31

**Authors:** Fuyong Bian, Yi Jin, Shaoming Chi, Guojun Shi, Sichuan Xu

**Affiliations:** a College of Chemical Science and Technology, Yunnan University, Kunming City, People’s Republic of China

## Abstract

In the crystal structure of the title compound, C_19_H_20_FN_2_O_3_^+^·C_2_F_3_O_2_^−^, N—H⋯O and O—H⋯O hydrogen bonds link two cations and two anions into a 22-atom ring. These rings are further linked into a three dimensional network by weak C—H⋯O contacts.

## Related literature

For the preparation for the title compound, see: Hoff *et al.* (2005 ▶); Wallén *et al.* (2003 ▶); Stachulski *et al.* (2006 ▶). For similar structures, see: Luedtkea & Mach (2003 ▶); Rok *et al.* (2007 ▶); Friedel & Crafts (1932*a* ▶,*b* ▶). For the applications of similar compounds, see: Wise (1996 ▶).
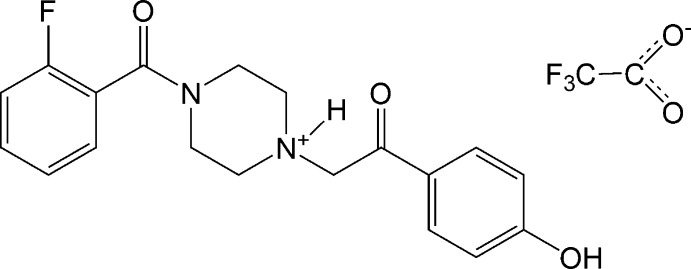


## Experimental

### 

#### Crystal data

C_19_H_20_FN_2_O_3_^+^·C_2_F_3_O_2_^−^
*M* *_r_* = 456.39
Monoclinic, 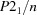

*a* = 10.055 (3) Å
*b* = 9.601 (2) Å
*c* = 21.946 (5) Å
β = 91.960 (4)°
*V* = 2117.4 (9) Å^3^
*Z* = 4
Mo *K*α radiation
μ = 0.13 mm^−1^
*T* = 298 K
0.23 × 0.20 × 0.18 mm

#### Data collection

Bruker SMART CCD area-detector diffractometer
Absorption correction: multi-scan (*SADABS*; Bruker, 2001 ▶) *T*_min_ = 0.956, *T*_max_ = 0.971
12540 measured reflections
3824 independent reflections
1534 reflections with *I* > 2σ(*I*)
*R*_int_ = 0.070

#### Refinement

*R*[*F* ^2^ > 2σ(*F* ^2^)] = 0.073
*wR*(*F* ^2^) = 0.240
*S* = 1.03
3824 reflections
289 parameters
H-atom parameters constrained
Δρ_max_ = 0.26 e Å^−3^
Δρ_min_ = −0.20 e Å^−3^

### 

Data collection: *SMART* (Bruker, 2001 ▶); cell refinement: *SAINT* (Bruker, 2001 ▶); data reduction: *SAINT*; program(s) used to solve structure: *SHELXS97* (Sheldrick, 2008 ▶); program(s) used to refine structure: *SHELXL97* (Sheldrick, 2008 ▶); molecular graphics: *SHELXTL* (Sheldrick, 2008 ▶); software used to prepare material for publication: *SHELXTL*.

## Supplementary Material

Crystal structure: contains datablock(s) I, global. DOI: 10.1107/S1600536812042857/qm2083sup1.cif

Structure factors: contains datablock(s) I. DOI: 10.1107/S1600536812042857/qm2083Isup2.hkl

Additional supplementary materials:  crystallographic information; 3D view; checkCIF report

## Figures and Tables

**Table 1:** Hydrogen-bond geometry (Å, °)

*D*—H⋯*A*	*D*—H	H⋯*A*	*D*⋯*A*	*D*—H⋯*A*
N1—H1*N*⋯O5^i^	0.91	1.87	2.710 (5)	152
O1—H1⋯O5^ii^	0.82	1.95	2.697 (5)	151
C8—H8*B*⋯O3^i^	0.97	2.22	2.996 (6)	136

Symmetry codes: (i) 
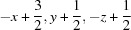
; (ii) 
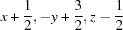
.

**Fig. 1.**
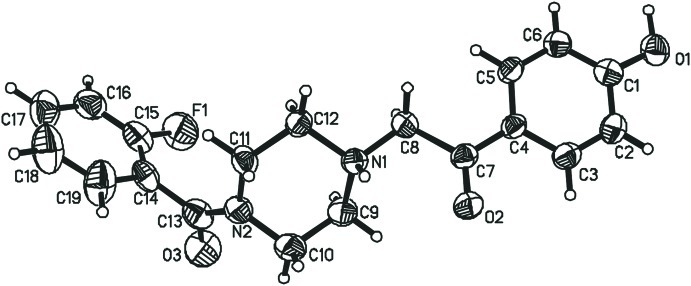
Ellipsoid plot

## supplementary crystallographic information

### Experimental

The title compound was obtained through the following four steps: 2-fluorobenzoic acid (700 mg, 5 mmol) was dissolved into 3 ml of SOCl_2_, the mixture stirred at 1033 K for 5 h and then slowly cooled to room temperature before removing excess SOCl_2_. After preparing an ethanol solution (10 ml) of 2-fluorobenzoyl chloride (317 mg, 2 mmol) and 1-boc-piperazine (372 mg, 4 mmol), potassium carbonate (552 mg, 4 mmol) was added to the solution. The mixture was refluxed for 12 h and then cooled to room temperature; extraction and concentration were performed to obtain an oil. After mixing the oil (a dichloromethane solution, 10 ml) with TFA (1.5 ml) and stirring for 12 h to complete the reaction, the mixture was poured into water. The aqueous layer was extracted by ethyl acetate, and then the organic layer was separated, dried using sodium sulfate to obtain 4-(2-fluorobenzoyl)piperazin-1-ium trifluoroacetate. Potassium carbonate (276 mg, 2 mmol) was added to the prepared ethanol solution (10 ml) of (2-fluorophenyl)(piperazin-1-yl)methanone (208 mg, 1 mmol) and 2-chloro-1-(4-hydroxyphenyl)ethanone (341 mg, 2 mmol), which was refluxed for 5 h and mixed by adding water. Again the aqueous layer was extracted by ethyl acetate for the separation of the organic layer, washed using anhydrous ethyl acetate, and dried using sodium sulfate, filtered, and concentrated in vacuum. The purification of residue by using silica gel column chromatography and eluting with EtOAc–petroleum ether (1:1) produced the pale-yellow solid (yield: 164 mg, 48%) of 4-(2-fluorobenzoyl)-1-[2-(4-hydroxyphenyl)-2-oxoethyl]piperazin-1-ium trifluoroacetate.

### Refinement

Hatomswereplacedincalculatedpositions[C—H=0.93(aromatic) and O—H= 0.82 or0.96Å (methylgroup)]and refined using a ridingmodel approximation with*U*_iso_(H) constrainedto1.2 (aromatic) or 1.5(methyl,O—H)times*U*_eq_ of therespectiveparentatom.

### Crystal data

**Table:**

C_19_H_20_FN_2_O_3_^+^·C_2_F_3_O_2_^−^	*F*(000) = 944
*M**_r_* = 456.39	*D*_x_ = 1.432 Mg m^−^^3^
Monoclinic, *P*2_1_/*n*	Mo *K*α radiation, λ = 0.71073 Å
*a* = 10.055 (3) Å	Cell parameters from 892 reflections
*b* = 9.601 (2) Å	θ = 2.3–17.0°
*c* = 21.946 (5) Å	µ = 0.13 mm^−^^1^
β = 91.960 (4)°	*T* = 298 K
*V* = 2117.4 (9) Å^3^	Parallelepiped, colourless
*Z* = 4	0.23 × 0.20 × 0.18 mm

### Data collection

**Table:**

Bruker SMART CCD area-detector diffractometer	3824 independent reflections
Radiation source: fine-focus sealed tube	1534 reflections with *I* > 2σ(*I*)
Graphite monochromator	*R*_int_ = 0.070
phi and ω scans	θ_max_ = 25.4°, θ_min_ = 1.9°
Absorption correction: multi-scan (*SADABS*; Bruker, 2001)	*h* = −12→12
*T*_min_ = 0.956, *T*_max_ = 0.971	*k* = 0→11
12540 measured reflections	*l* = 0→16

### Refinement

**Table:**

Refinement on *F*^2^	Primary atom site location: structure-invariant direct methods
Least-squares matrix: full	Secondary atom site location: difference Fourier map
*R*[*F*^2^ > 2σ(*F*^2^)] = 0.073	Hydrogen site location: inferred from neighbouring sites
*wR*(*F*^2^) = 0.240	H-atom parameters constrained
*S* = 1.03	*w* = 1/[σ^2^(*F*_o_^2^) + (0.1*P*)^2^] where *P* = (*F*_o_^2^ + 2*F*_c_^2^)/3
3824 reflections	(Δ/σ)_max_ < 0.001
289 parameters	Δρ_max_ = 0.26 e Å^−^^3^
0 restraints	Δρ_min_ = −0.20 e Å^−^^3^

### Special details

**Table:**

Geometry. All esds (except the esd in the dihedral angle between two l.s. planes) are estimated using the full covariance matrix. The cell esds are taken into account individually in the estimation of esds in distances, angles and torsion angles; correlations between esds in cell parameters are only used when they are defined by crystal symmetry. An approximate (isotropic) treatment of cell esds is used for estimating esds involving l.s. planes.
Refinement. Refinement of F^2^ against ALL reflections. The weighted R-factor wR and goodness of fit S are based on F^2^, conventional R-factors R are based on F, with F set to zero for negative F^2^. The threshold expression of F^2^ > 2sigma(F^2^) is used only for calculating R-factors(gt) etc. and is not relevant to the choice of reflections for refinement. R-factors based on F^2^ are statistically about twice as large as those based on F, and R- factors based on ALL data will be even larger.

### Fractional atomic coordinates and isotropic or equivalent isotropic displacement parameters (Å^2^)

**Table:**

	*x*	*y*	*z*	*U*_iso_*/*U*_eq_	
F1	0.6641 (4)	0.7921 (4)	0.35902 (18)	0.1420 (13)	
F2	−0.0352 (4)	0.0629 (4)	0.3531 (2)	0.1733 (18)	
F3	−0.0010 (4)	0.2786 (4)	0.35550 (19)	0.1465 (15)	
F4	−0.0132 (4)	0.1720 (4)	0.27235 (19)	0.1558 (16)	
N1	0.9794 (3)	0.7317 (3)	0.17362 (14)	0.0623 (9)	
H1N	1.0634	0.7032	0.1657	0.081*	
N2	0.9521 (4)	0.6187 (4)	0.29247 (17)	0.0772 (10)	
O1	0.7573 (3)	0.9834 (3)	−0.15804 (14)	0.0945 (11)	
H1	0.7304	1.0632	−0.1533	0.123*	
O2	1.0042 (3)	0.6279 (3)	0.05966 (14)	0.0850 (9)	
O3	0.7802 (4)	0.5059 (4)	0.33367 (18)	0.1197 (13)	
O4	0.2142 (4)	0.0147 (4)	0.3220 (2)	0.1226 (14)	
O5	0.2540 (3)	0.2358 (4)	0.34091 (16)	0.0951 (11)	
C1	0.7985 (4)	0.9296 (5)	−0.1033 (2)	0.0742 (12)	
C2	0.8442 (5)	0.7938 (5)	−0.1037 (2)	0.0813 (13)	
H2	0.8444	0.7442	−0.1401	0.106*	
C3	0.8891 (4)	0.7321 (4)	−0.0505 (2)	0.0753 (12)	
H3	0.9200	0.6409	−0.0512	0.098*	
C4	0.8891 (4)	0.8047 (4)	0.00491 (19)	0.0642 (11)	
C5	0.8382 (4)	0.9385 (4)	0.0040 (2)	0.0720 (12)	
H5	0.8336	0.9874	0.0405	0.094*	
C6	0.7943 (4)	1.0014 (5)	−0.0493 (2)	0.0766 (12)	
H6	0.7618	1.0921	−0.0488	0.100*	
C7	0.9441 (4)	0.7384 (4)	0.06037 (19)	0.0653 (11)	
C8	0.9287 (5)	0.8139 (4)	0.12025 (18)	0.0711 (12)	
H8A	0.9763	0.9016	0.1190	0.092*	
H8B	0.8353	0.8346	0.1253	0.092*	
C9	0.8978 (5)	0.6055 (4)	0.1846 (2)	0.0846 (14)	
H9A	0.8992	0.5449	0.1493	0.110*	
H9B	0.8063	0.6325	0.1908	0.110*	
C10	0.9516 (5)	0.5284 (4)	0.2399 (2)	0.0872 (14)	
H10A	0.8969	0.4473	0.2472	0.113*	
H10B	1.0415	0.4968	0.2329	0.113*	
C11	1.0351 (5)	0.7411 (4)	0.28498 (18)	0.0728 (12)	
H11A	1.1268	0.7127	0.2803	0.095*	
H11B	1.0314	0.7994	0.3210	0.095*	
C12	0.9874 (4)	0.8226 (4)	0.22957 (18)	0.0684 (11)	
H12A	0.9003	0.8614	0.2368	0.089*	
H12B	1.0480	0.8993	0.2228	0.089*	
C13	0.8623 (5)	0.6010 (6)	0.3364 (2)	0.0864 (14)	
C14	0.8670 (5)	0.6952 (5)	0.3901 (2)	0.0782 (13)	
C15	0.7657 (6)	0.7849 (6)	0.4023 (3)	0.0935 (15)	
C16	0.7596 (7)	0.8661 (6)	0.4533 (3)	0.1098 (19)	
H16	0.6881	0.9251	0.4598	0.143*	
C17	0.8654 (8)	0.8552 (7)	0.4944 (3)	0.117 (2)	
H17	0.8663	0.9095	0.5295	0.152*	
C18	0.9690 (7)	0.7671 (8)	0.4851 (3)	0.133 (2)	
H18	1.0400	0.7609	0.5133	0.173*	
C19	0.9659 (6)	0.6875 (7)	0.4329 (3)	0.124 (2)	
H19	1.0354	0.6255	0.4269	0.161*	
C20	0.1836 (5)	0.1356 (6)	0.3310 (2)	0.0810 (14)	
C21	0.0321 (5)	0.1635 (6)	0.3290 (3)	0.0932 (15)	

### Atomic displacement parameters (Å^2^)

**Table:**

	*U*^11^	*U*^22^	*U*^33^	*U*^12^	*U*^13^	*U*^23^
F1	0.138 (3)	0.151 (3)	0.135 (3)	0.049 (2)	−0.022 (2)	0.000 (2)
F2	0.103 (3)	0.157 (3)	0.262 (5)	−0.037 (2)	0.036 (3)	0.053 (3)
F3	0.120 (3)	0.135 (3)	0.185 (4)	0.039 (2)	0.010 (3)	−0.046 (3)
F4	0.137 (3)	0.195 (4)	0.131 (3)	0.028 (3)	−0.059 (3)	−0.024 (3)
N1	0.068 (2)	0.0591 (19)	0.060 (2)	−0.0027 (16)	0.0064 (16)	0.0012 (15)
N2	0.089 (3)	0.071 (2)	0.073 (2)	−0.011 (2)	0.016 (2)	0.0063 (19)
O1	0.112 (3)	0.094 (2)	0.076 (2)	−0.0032 (18)	−0.0173 (19)	0.0037 (17)
O2	0.099 (2)	0.075 (2)	0.081 (2)	0.0236 (17)	0.0098 (17)	−0.0025 (16)
O3	0.113 (3)	0.121 (3)	0.127 (3)	−0.045 (2)	0.034 (2)	0.004 (2)
O4	0.106 (3)	0.093 (3)	0.169 (4)	0.002 (2)	0.004 (3)	−0.007 (3)
O5	0.088 (2)	0.093 (2)	0.104 (3)	−0.0177 (19)	0.0044 (19)	−0.0028 (19)
C1	0.072 (3)	0.071 (3)	0.079 (3)	−0.004 (2)	−0.009 (2)	0.008 (2)
C2	0.092 (3)	0.086 (3)	0.066 (3)	0.002 (3)	−0.008 (3)	−0.006 (2)
C3	0.078 (3)	0.067 (3)	0.082 (3)	−0.001 (2)	0.013 (2)	−0.009 (2)
C4	0.067 (3)	0.059 (2)	0.067 (3)	−0.003 (2)	0.003 (2)	−0.011 (2)
C5	0.079 (3)	0.064 (3)	0.072 (3)	0.000 (2)	0.001 (2)	−0.009 (2)
C6	0.076 (3)	0.073 (3)	0.080 (3)	−0.001 (2)	−0.002 (2)	0.000 (2)
C7	0.065 (3)	0.060 (3)	0.072 (3)	−0.005 (2)	0.011 (2)	−0.005 (2)
C8	0.087 (3)	0.063 (2)	0.063 (3)	0.012 (2)	−0.001 (2)	−0.003 (2)
C9	0.097 (3)	0.068 (3)	0.088 (3)	−0.030 (3)	0.010 (3)	−0.002 (2)
C10	0.104 (4)	0.062 (3)	0.096 (4)	−0.016 (2)	0.017 (3)	0.010 (3)
C11	0.081 (3)	0.077 (3)	0.061 (3)	−0.016 (2)	0.001 (2)	0.010 (2)
C12	0.077 (3)	0.064 (2)	0.064 (3)	−0.004 (2)	0.004 (2)	−0.004 (2)
C13	0.083 (3)	0.085 (3)	0.092 (4)	−0.005 (3)	0.012 (3)	0.019 (3)
C14	0.068 (3)	0.095 (3)	0.073 (3)	−0.007 (3)	0.013 (2)	0.026 (3)
C15	0.090 (4)	0.099 (4)	0.091 (4)	0.010 (3)	0.003 (3)	0.029 (3)
C16	0.148 (6)	0.087 (4)	0.097 (4)	0.010 (4)	0.034 (4)	0.014 (3)
C17	0.140 (6)	0.142 (6)	0.070 (4)	−0.021 (5)	0.015 (4)	0.003 (3)
C18	0.095 (5)	0.203 (7)	0.102 (5)	0.026 (5)	0.004 (4)	0.006 (5)
C19	0.095 (4)	0.185 (6)	0.092 (4)	0.025 (4)	0.014 (4)	−0.026 (4)
C20	0.105 (4)	0.070 (3)	0.067 (3)	−0.012 (3)	0.000 (3)	0.003 (2)
C21	0.078 (3)	0.098 (4)	0.104 (4)	0.001 (3)	−0.001 (3)	0.000 (3)

### Geometric parameters (Å, º)

**Table:**

F1—C15	1.372 (6)	C6—H6	0.9300
F2—C21	1.301 (6)	C7—C8	1.513 (5)
F3—C21	1.298 (6)	C8—H8A	0.9700
F4—C21	1.312 (6)	C8—H8B	0.9700
N1—C8	1.488 (5)	C9—C10	1.505 (6)
N1—C9	1.488 (5)	C9—H9A	0.9700
N1—C12	1.506 (5)	C9—H9B	0.9700
N1—H1N	0.9100	C10—H10A	0.9700
N2—C13	1.353 (6)	C10—H10B	0.9700
N2—C10	1.443 (5)	C11—C12	1.510 (5)
N2—C11	1.454 (5)	C11—H11A	0.9700
O1—C1	1.360 (5)	C11—H11B	0.9700
O1—H1	0.8200	C12—H12A	0.9700
O2—C7	1.221 (4)	C12—H12B	0.9700
O3—C13	1.231 (5)	C13—C14	1.486 (7)
O4—C20	1.219 (5)	C14—C19	1.346 (7)
O5—C20	1.209 (5)	C14—C15	1.368 (7)
C1—C6	1.373 (6)	C15—C16	1.367 (7)
C1—C2	1.382 (6)	C16—C17	1.375 (8)
C2—C3	1.372 (6)	C16—H16	0.9300
C2—H2	0.9300	C17—C18	1.363 (8)
C3—C4	1.402 (6)	C17—H17	0.9300
C3—H3	0.9300	C18—C19	1.377 (8)
C4—C5	1.383 (5)	C18—H18	0.9300
C4—C7	1.465 (6)	C19—H19	0.9300
C5—C6	1.376 (6)	C20—C21	1.546 (7)
C5—H5	0.9300		
			
C8—N1—C9	112.6 (3)	N2—C10—H10B	109.7
C8—N1—C12	110.0 (3)	C9—C10—H10B	109.7
C9—N1—C12	110.7 (3)	H10A—C10—H10B	108.2
C8—N1—H1N	107.8	N2—C11—C12	110.0 (3)
C9—N1—H1N	107.8	N2—C11—H11A	109.7
C12—N1—H1N	107.8	C12—C11—H11A	109.7
C13—N2—C10	120.7 (4)	N2—C11—H11B	109.7
C13—N2—C11	125.5 (4)	C12—C11—H11B	109.7
C10—N2—C11	112.4 (3)	H11A—C11—H11B	108.2
C1—O1—H1	109.5	N1—C12—C11	111.3 (3)
O1—C1—C6	123.7 (4)	N1—C12—H12A	109.4
O1—C1—C2	116.3 (4)	C11—C12—H12A	109.4
C6—C1—C2	120.0 (4)	N1—C12—H12B	109.4
C3—C2—C1	120.1 (4)	C11—C12—H12B	109.4
C3—C2—H2	120.0	H12A—C12—H12B	108.0
C1—C2—H2	120.0	O3—C13—N2	121.4 (5)
C2—C3—C4	121.0 (4)	O3—C13—C14	119.5 (5)
C2—C3—H3	119.5	N2—C13—C14	119.0 (5)
C4—C3—H3	119.5	C19—C14—C15	115.9 (5)
C5—C4—C3	117.4 (4)	C19—C14—C13	121.5 (5)
C5—C4—C7	123.0 (4)	C15—C14—C13	122.3 (5)
C3—C4—C7	119.6 (4)	C16—C15—C14	125.0 (6)
C6—C5—C4	121.9 (4)	C16—C15—F1	119.0 (6)
C6—C5—H5	119.1	C14—C15—F1	116.0 (5)
C4—C5—H5	119.1	C15—C16—C17	116.0 (6)
C1—C6—C5	119.7 (4)	C15—C16—H16	122.0
C1—C6—H6	120.2	C17—C16—H16	122.0
C5—C6—H6	120.2	C18—C17—C16	121.8 (6)
O2—C7—C4	122.7 (4)	C18—C17—H17	119.1
O2—C7—C8	119.5 (4)	C16—C17—H17	119.1
C4—C7—C8	117.8 (4)	C17—C18—C19	118.2 (6)
N1—C8—C7	112.8 (3)	C17—C18—H18	120.9
N1—C8—H8A	109.0	C19—C18—H18	120.9
C7—C8—H8A	109.0	C14—C19—C18	123.0 (6)
N1—C8—H8B	109.0	C14—C19—H19	118.5
C7—C8—H8B	109.0	C18—C19—H19	118.5
H8A—C8—H8B	107.8	O5—C20—O4	129.6 (6)
N1—C9—C10	110.3 (4)	O5—C20—C21	116.0 (5)
N1—C9—H9A	109.6	O4—C20—C21	114.5 (5)
C10—C9—H9A	109.6	F3—C21—F2	107.8 (5)
N1—C9—H9B	109.6	F3—C21—F4	106.6 (5)
C10—C9—H9B	109.6	F2—C21—F4	105.3 (5)
H9A—C9—H9B	108.1	F3—C21—C20	113.7 (5)
N2—C10—C9	109.9 (4)	F2—C21—C20	112.7 (5)
N2—C10—H10A	109.7	F4—C21—C20	110.3 (5)
C9—C10—H10A	109.7		
			
O1—C1—C2—C3	179.2 (4)	N2—C11—C12—N1	−54.0 (5)
C6—C1—C2—C3	−2.0 (7)	C10—N2—C13—O3	1.8 (7)
C1—C2—C3—C4	0.3 (7)	C11—N2—C13—O3	167.4 (4)
C2—C3—C4—C5	2.1 (6)	C10—N2—C13—C14	179.3 (4)
C2—C3—C4—C7	−176.9 (4)	C11—N2—C13—C14	−15.1 (7)
C3—C4—C5—C6	−2.8 (6)	O3—C13—C14—C19	106.7 (6)
C7—C4—C5—C6	176.1 (4)	N2—C13—C14—C19	−70.8 (6)
O1—C1—C6—C5	−180.0 (4)	O3—C13—C14—C15	−66.8 (7)
C2—C1—C6—C5	1.4 (7)	N2—C13—C14—C15	115.7 (5)
C4—C5—C6—C1	1.1 (7)	C19—C14—C15—C16	0.8 (8)
C5—C4—C7—O2	−170.3 (4)	C13—C14—C15—C16	174.7 (5)
C3—C4—C7—O2	8.6 (6)	C19—C14—C15—F1	−179.4 (5)
C5—C4—C7—C8	7.6 (6)	C13—C14—C15—F1	−5.6 (7)
C3—C4—C7—C8	−173.5 (4)	C14—C15—C16—C17	0.6 (8)
C9—N1—C8—C7	−67.6 (4)	F1—C15—C16—C17	−179.1 (5)
C12—N1—C8—C7	168.4 (3)	C15—C16—C17—C18	−1.0 (9)
O2—C7—C8—N1	−6.2 (6)	C16—C17—C18—C19	0.0 (10)
C4—C7—C8—N1	175.8 (3)	C15—C14—C19—C18	−1.9 (9)
C8—N1—C9—C10	−178.6 (4)	C13—C14—C19—C18	−175.8 (6)
C12—N1—C9—C10	−55.1 (5)	C17—C18—C19—C14	1.6 (11)
C13—N2—C10—C9	106.7 (5)	O5—C20—C21—F3	−19.3 (7)
C11—N2—C10—C9	−60.7 (5)	O4—C20—C21—F3	160.9 (5)
N1—C9—C10—N2	58.3 (5)	O5—C20—C21—F2	−142.4 (5)
C13—N2—C11—C12	−108.2 (5)	O4—C20—C21—F2	37.8 (7)
C10—N2—C11—C12	58.4 (5)	O5—C20—C21—F4	100.3 (5)
C8—N1—C12—C11	178.4 (3)	O4—C20—C21—F4	−79.5 (6)
C9—N1—C12—C11	53.4 (5)		

### Hydrogen-bond geometry (Å, º)

**Table:**

*D*—H···*A*	*D*—H	H···*A*	*D*···*A*	*D*—H···*A*
N1—H1*N*···O5^i^	0.91	1.87	2.710 (5)	152
O1—H1···O5^ii^	0.82	1.95	2.697 (5)	151
C8—H8*B*···O3^i^	0.97	2.22	2.996 (6)	136

Symmetry codes: (i) −*x*+3/2, *y*+1/2, −*z*+1/2; (ii) *x*+1/2, −*y*+3/2, *z*−1/2.
